# Scattering‐Enhanced Light Extraction for Radiative Thermal Load Mitigation in Fluorescent Films

**DOI:** 10.1002/advs.202510643

**Published:** 2025-09-15

**Authors:** Chenglong She, Yi Zhang, Minghao Dong, Xiaopeng Bai, Chenxi Wang, Fan Yang, Xiaobo Yin

**Affiliations:** ^1^ Department of Mechanical Engineering The University of Hong Kong Hong Kong 999077 China; ^2^ Department of Physics The University of Hong Kong Hong Kong 999077 China; ^3^ State Key Laboratory of Optical Quantum Materials The University of Hong Kong Hong Kong 999077 China; ^4^ Kadoorie Centre The University of Hong Kong Hong Kong 999077 China

**Keywords:** color, fluorescence, heat, light extraction, scattering

## Abstract

To mitigate solar heating in colored objects, fluorescent coloration has been proposed as an alternative to traditional absorptive pigments. However, the Stokes‐shifted photons generated by fluorophores predominantly remain trapped by total internal reflection (TIR), increasing the parasitic solar absorption and the radiative thermal load. This work introduces a scattering‐enhanced light extraction strategy that overcomes the TIR limit in fluorescent films. A sequential quadratic programming‐driven optimization model establishes the theoretical minimum radiative thermal load for both traditional and fluorescent‐colored surfaces. Results reveal that while traditional absorption‐based color achieves only 19.3% sub‐ambient cooling chromaticity in the CIE 1931 color space, light extraction technology expands the range from 26.7% to 64.9% for fluorescent color. TiO_2_ nanoparticles enhance light extraction through multiple Mie‐scattering, with Monte Carlo ray‐tracing simulation identifying an optimal 0.5 wt% TiO_2_ nanoparticle concentration yielding 85.9% light extraction efficiency, significantly outperforming the TiO_2_‐free fluorescent film (25.3%) and a higher 15 wt.% concentration (66.6%). Outdoor experiments confirm the optimal 0.5 wt% sample exhibits a 4.1 °C temperature decrease compared to the control (0 wt.%) sample. This approach offers cost‐effective scalability advantages over microtexture‐based light extraction methods.

## Graphical abstract

Scattering‐enhanced light extraction overcomes total internal reflection limitations in fluorescent films, expanding the sub‐ambient cooling color range from 26.7% to 64.9% in CIE 1931 space. Optimal TiO_2_ nanoparticle doping (0.5 wt.%) achieves 85.9% light extraction efficiency and 89.5% visible reflectance. Outdoor experiments demonstrate 4.1 °C temperature reduction compared to undoped films, offering scalable colored cooling solutions.

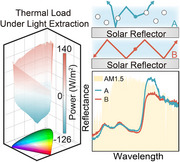

## 1 Introduction

Global cooling energy demand is projected to triple by 2050, driving urgent efforts to optimize radiative thermal load (radiative heat exchange between objects, sun, and environment).^[^ ^1^, ^2^ ^]^ High‐load surfaces (e.g., brown roofs absorbing 80–90% sunlight) can be 14 °C hotter than their low‐load counterparts.^[^ ^3^ ^]^ Conventional cooling materials reduce heat via high solar reflectance but impose aesthetic limitations (e.g., white/silver hues causing glare).^[^ ^4^, ^5^, ^6^, ^7^, ^8^, ^9^, ^10^, ^11^, ^12^, ^13^ ^]^ Expanding color diversity while retaining thermal performance remains critical for sustainable urban cooling. Three mechanisms have been utilized to lower the radiative thermal load of colored objects. The first approach employs dyes that selectively absorb specific visible wavelengths due to their intrinsic absorptive properties.^[^ ^14^, ^15^, ^16^ ^]^ The second method achieves structural coloration through structures, such as multi‐layer configurations,^[^ ^17^, ^18^, ^19^ ^]^ periodic surface structures,^[^ ^20^, ^21^, ^22^ ^]^ 3D photonic crystals,^[^ ^23^, ^24^ ^]^ core‐shell nanoparticles,^[^ ^25^ ^]^ and plasmonic structures.^[^ ^26^, ^27^ ^]^ However, existing approaches either rely on broadband absorption (compromising cooling performance) or narrowband reflection (yielding low‐saturation hues), collectively limiting the color gamut.

The third approach utilizes photoluminescent materials to generate coloration. Unlike the previous two methods, photoluminescence (PL) absorbs specific photons but re‐emits photons at different wavelengths, thereby partially mitigating heat generation. However, total internal reflection (TIR) causes significant light trapping, which limits radiative thermal load reduction in fluorescent objects. Consequently, efficient extraction of re‐emitted light has become fundamental to ensuring cooling performance while maintaining distinct color. Light extraction (LE) efficiency has been extensively investigated in various domains. For instance, researchers have modified LED bead geometries or chip surface morphologies to enhance light output,^[^ ^28^, ^29^ ^]^ designed optical cavities within internal light‐emitting regions to augment spontaneous emission.^[^ ^30^, ^31^ ^]^ Meanwhile, a refined microstructure strategy is applied on fluorescent film surfaces, enabling increased photosynthetic productivity and biomass generation.^[^ ^32^, ^33^ ^]^ However, the high costs associated with large‐scale manufacturing of these structures and potential structural degradation under extended outdoor exposure conditions are still significant challenges.

In this study, we theoretically calculated the radiative thermal load modifications in fluorescent films across the entire CIE 1931 color space under varying LE efficiencies. At the theoretical limit of 100% LE efficiency, the critical color range for which radiative thermal load equals zero expanded from 19.3% to 64.9%, demonstrating that enhanced LE can significantly broaden the color gamut of fluorescent films while maintaining cooling performance. Through simulation, we implemented a scattering‐enhanced LE strategy. By incorporating 0.5 wt% TiO2 nanoparticles into polymer film encapsulating fluorescent powders, the LE efficiency can increase from 25.3% in the undoped control to an optimized 85.9%, whereas a higher concentration (15 wt%) yields a reduced efficiency of 66.6%. We then fabricated three samples with corresponding dopant concentrations of 0, 0.5, and 15 wt%. With increasing TiO2 dopant concentration, the color lightness of the fluorescent films exhibits a monotonic enhancement (47%, 56%, and 65% for 0, 0.5, and 15 wt%, respectively). Interestingly, the total solar reflectance exhibited non‐monotonic behavior, initially increasing from 83.1% (undoped) to a maximum of 89.5% at optimal doping (0.5 wt%) before decreasing to 86.2% at the highest concentration (15 wt%). Outdoor temperature measurements revealed that film with 0.5 wt% TiO2 doping was up to 4.1 °C lower than the undoped film, and 2.0 °C lower than the highly doped film with higher lightness coloration.

## 2 Results

### 2.1 Scattering‐Enhanced Light Extraction

Due to metamerism, objects with identical perceived colors can exhibit substantially different visible reflectance profiles. Consequently, significant radiative thermal load variations may exist between them due to differential solar absorption. The visible reflectance spectrum establishes the connection between color perception and radiative thermal load. Li, et al.^[^ ^17^ ^]^ previously established a quantitative relationship between color and radiative thermal load in outdoor objects. For a chosen color, determining the extremal radiative thermal load requires optimization algorithms to resolve such a nonlinear‐constrained programming problem. However, that analysis was restricted to non‐photoluminescent (Non‐PL) coloration mechanisms. While Wang, et al. and Min, et al.^[^ ^34^, ^35^ ^]^ have subsequently advanced calculation methodologies that incorporate PL with quantum efficiency, these frameworks did not adequately discuss the role of light extraction (LE) efficiency in determining the overall thermal performance of fluorescent systems.

**Figure 1:**
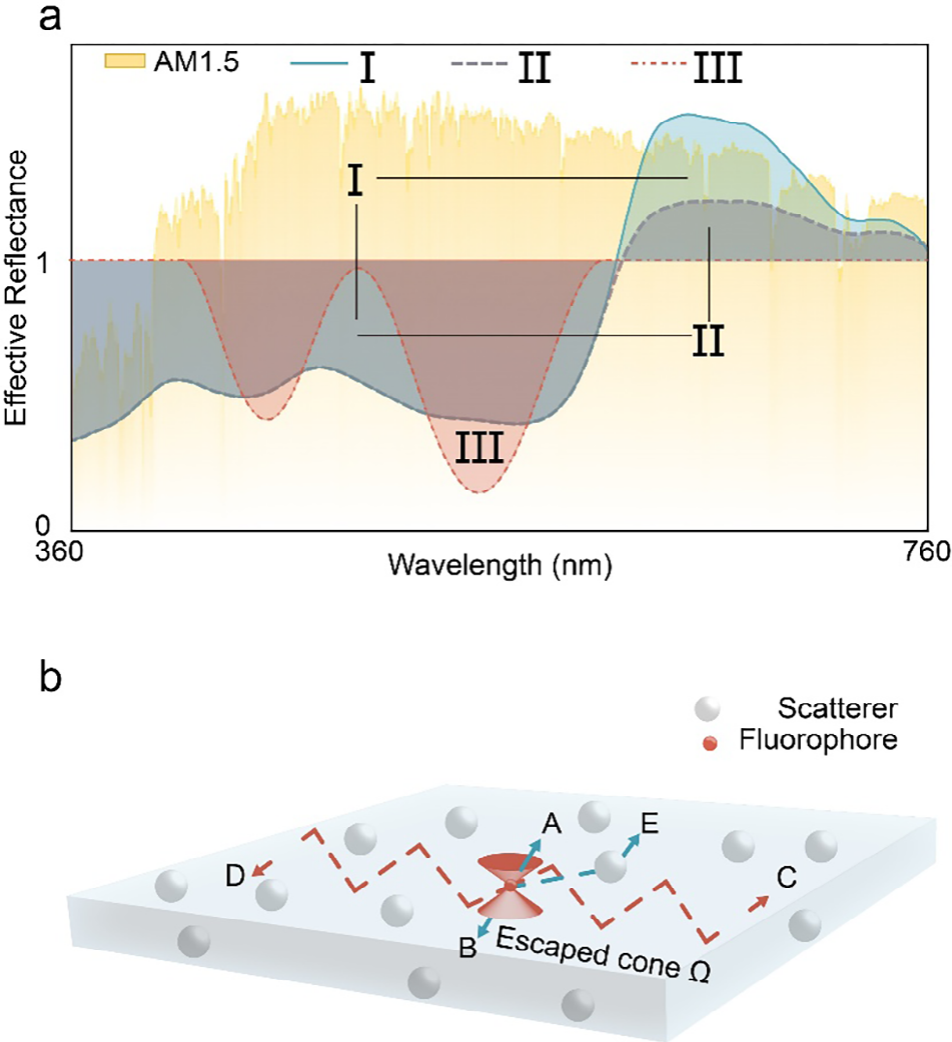
The schematic diagram of light extraction (LE) enhancement via a scattering medium. a) Schematic comparison of effective visible reflectance (360–760 nm) of two fluorescent films (I: high LE efficiency; II: low LE efficiency) and a non‐fluorescent film III (color‐match to I). Despite identical absorption (shared shaded region), film I exhibits a higher reflectance peak than II due to higher LE. Non‐PL film III matches the total visible reflectance of film I, despite differing absorption, underscoring LE's role in modulating reflectance despite comparable color. b) Illustration of TIR limitation in a planar polymeric film containing one fluorophore. Ray A and B within the escape cone Ω can directly exit the film, while rays C and D become trapped due to TIR. Random light scattering events can redirect trapped light (ray E) into the escape cone, enhancing LE efficiency.

**Figure** **1**a schematically illustrates the comparison among visible reflectance spectral profiles (360–760 nm) of two fluorescent films (I with high LE efficiency and II with low LE efficiency) and a Non‐PL film (III, same color as I). The PL process generates energy contribution in the reflection direction from re‐emitted light, augmenting the reflectance, potentially exceeding 100%. Consequently, “effective reflectance”^[^ ^35^, ^36^, ^37^ ^]^ is employed to characterize the reflective energy distribution in this scenario. Even when two fluorescent films exhibit identical absorption (common shadow area of I and II in Figure 1a), differences in reflection peak intensity arise due to LE effects. Compared to film II, film I produces a lighter color appearance and higher visible reflectance. Moreover, compared to Non‐PL film III with the same color but less absorption, fluorescent film I with high LE efficiency has the same total visible reflectance. To quantify how the LE effect affects the radiative thermal load of fluorescent film, we analyzed three scenarios: Non‐PL, 25%, and 100% LE efficiency, employing a sequential quadratic programming‐driven radiative thermal load optimization model considering the photon recycling process to determine the minimum radiative thermal load across the CIE 1931 color space. The comprehensive computational workflow incorporating spectral‐conversion energy loss, redistribution processes, and optimization algorithms is detailed in Supporting Information Section 1, with results presented in the subsequent section.

The LE efficiency of a planar polymeric film encapsulating fluorescent particles is limited by TIR, which occurs when light propagates from a high‐refractive‐index medium to a low‐index medium at angles exceeding the critical angle. As illustrated in Figure 1b, rays A and B within the escape cone Ω, defined by rotating the critical angle around the normal axis, can directly propagate into the low‐index medium, whereas rays C and D outside this cone become trapped within the film. Beyond surface structural modifications, exploitation of random light scattering can effectively alter light propagation paths, increasing the probability of rays re‐entering Ω, as exemplified by ray E, which initially bounces between top and bottom surfaces before entering the escape cone following scattering events. Note that only one fluorophore has been placed here for clear display.

### 2.2 Optimization of Radiative Thermal Load

Prior to investigating the influence of scatterers on LE efficiency, we theoretically calculated the impact of varying LE efficiencies on the minimum thermal load across the CIE 1931 color space. The radiative thermal load *P_thermal_* of an object under solar illumination is governed by its absorption and emission properties across the electromagnetic spectrum. The total radiative thermal load (W/m^2^) can be expressed as:

(1) $$P_{thermal}=P_{UV}+P_{VIS}+P_{NIR}-\left(P_{rad}-P_{atm}\right)$$

The absorbed solar power is partitioned into visible and near‐infrared absorption components, *P_VIS_* and *P_NIR_*. *P_rad_* and *P_atm_* denote the thermal emission of objects and the power absorbed from atmospheric radiation (Supporting Information Section 1.1). They are all related to the reflectance spectrum *r*(λ). However, to associate color coordinates with minimized *P_thermal_*, only visible reflectance contributes simultaneously to both color perception (Supporting Information Section 1.2) and thermal load. UV *P_UV_*, Near‐infrared absorption *P_NIR_* and net infrared thermal radiation (*P_rad_* − *P_atm_*) can be treated as constants (0 for *P_UV_* and *P_NIR_* and 130 W/m^2^ for *P_rad_* − *P_atm_*) for optimal thermal load calculations. Therefore, the visible reflectance spectrum *r*(λ) must simultaneously satisfy two criteria: 1. Minimize *P_VIS_* by maximizing visible reflectance *r*(λ). 2. Ensure the calculated CIE 1931 chromaticity coordinate (*x_cal_*,*y_cal_*) matches the target coordinate (*x_tar_*,*y_tar_*) within a tolerance of Δ, i.e., the chromaticity difference Δ*E* ≤ Δ. Thus, the problem transforms into a nonlinear‐constrained programming optimization problem with a defined objective function *P_VIS_*, chromaticity constraint Δ*E* ≤ Δ, and reflectance boundary constraint 0 ≤ *r*(λ) ≤ 1:

(2) $$\mathrm{minimize}\to P_{\mathrm{VIS}}=\int_{360\mathrm{nm}}^{760\mathrm{nm}}I_{\mathrm{AM}1.5}\left(\lambda \right)\cdot \left[1-r\left(\lambda \right)\right]d\lambda$$

(3) $$\mathrm{subject}\;\mathrm{to}\to \Delta E=\sqrt{{\left(x_{\mathrm{cal}}-x_{\mathrm{tar}}\right)}^{2}+{\left(y_{\mathrm{cal}}-y_{\mathrm{tar}}\right)}^{2}}\leq \Delta$$

(4) $$0\leq r\left(\lambda \right)\leq 1$$

**Figure 2:**
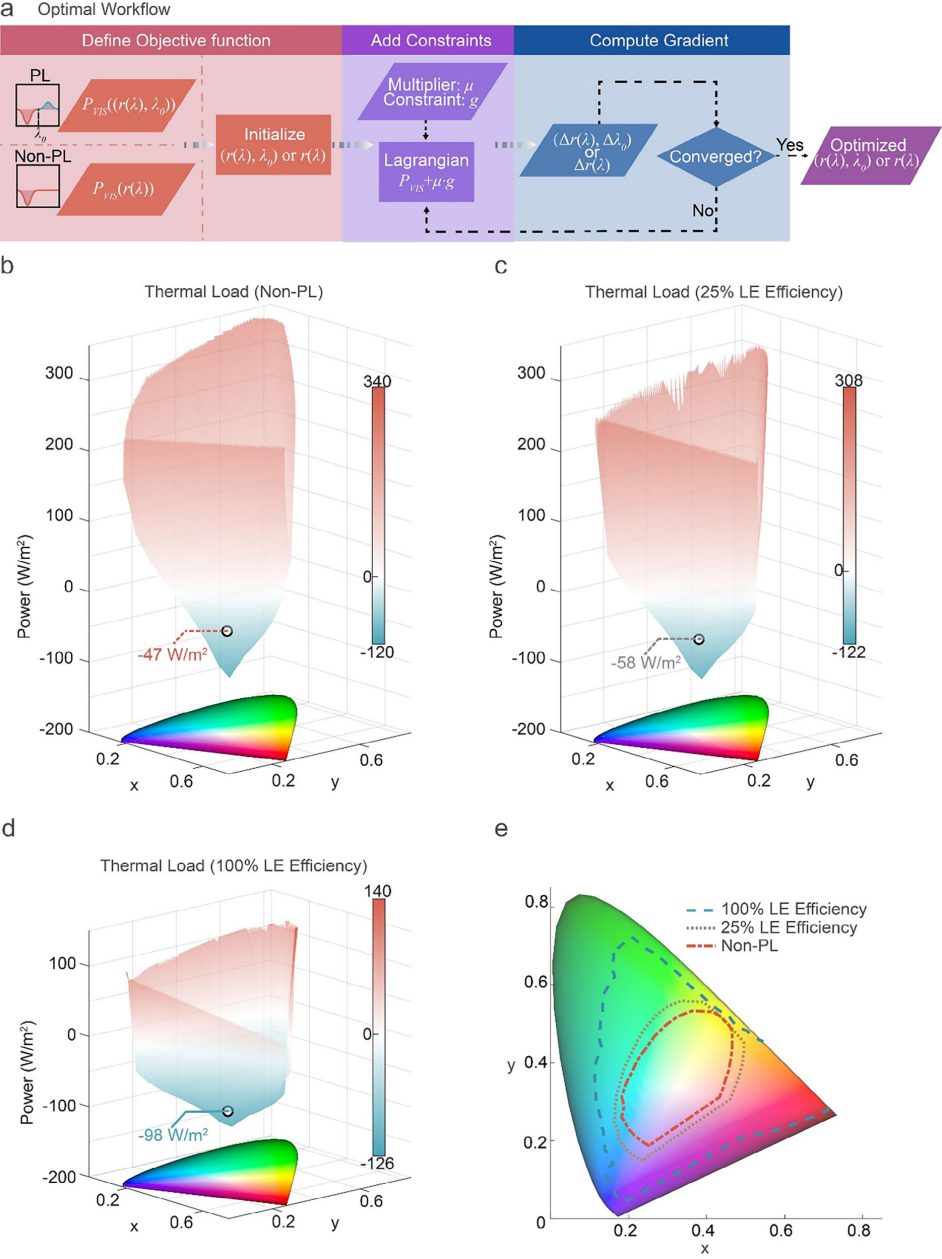
The theoretical results of the minimum radiative thermal load of different colors with different LE efficiency. a) Iterative optimization workflow for PL and Non‐PL color. The objective function incorporates visible reflectance *r*(λ), and absorption cut‐off wavelength λ_0_, (if PL is active). A Lagrangian function constrains the objective function *P_VIS_*, with Lagrange multiplier μ dynamically updated based on constraint violations. Variables are iteratively refined via gradient computation until convergence. 3D thermal load distribution surfaces for b) Non‐PL (LE = 0%), c) 25% LE efficiency, and d) 100% LE efficiency systems. Thermal loads span −120 to 340 W m^−2^ (Non‐PL), ‐122 to 308 W m^−2^ (25% LE), and ‐126 to 140 W m^−2^ (100% LE). At the target chromaticity (0.3728, 0.3029), thermal loads decrease from −47 W m^−2^ (Non‐PL) to −58 W m^−2^ (25% LE) and −98 W m^−2^ (100% LE). White boundaries denote zero thermal load, with red (positive) and teal (negative) regions indicating above‐ and sub‐ambient loads. Enhanced LE shifts the surface downward, reducing thermal load, expanding sub‐ambient color ranges. e) Projections of critical zero‐thermal‐load boundaries onto the CIE 1931 chromaticity plane for Non‐PL (red dashed line), 25% LE (gray dashed line), and 100% LE (teal dashed line) scenarios. Sub‐ambient regions occupy 19.3%, 26.7%, and 64.9% of the gamut, respectively, demonstrating LE efficiency's role in enabling passive cooling across broader color spaces.

The optimal workflow computational is depicted in **Figure** **2**a. The initialization of the objective function *P_VIS_* depends on whether the film undergoes the PL process. If PL occurs, the variable set will encompass not only the visible reflectance *r*(λ), but also the absorption cut‐off wavelength λ_0_ (Supporting Information Section 1.3). Subsequently, a Lagrangian function is constructed to constrain the objective function *P_VIS_* with constraint function *g*. The Lagrange multiplier *μ*, associated with these constraint functions is then automatically updated based on the degree of constraint violation. Finally, the gradient of the entire function is computed, enabling the iterative update of the variables. This iterative process continues until the objective function is assessed to have converged, at which point the optimal variable values are output.

To elucidate the contributions of the PL process and LE effect, Figure 2b–d depicts the optimal 3D thermal load distribution surfaces for films exhibiting Non‐PL, 25% LE efficiency, and 100% LE efficiency characteristics as functions of color coordinates. A target chromaticity coordinate of (0.3728, 0.3029), derived from the reflectance spectrum I in Figure 1a under high LE conditions, is highlighted for detailed analysis. Figure 2b illustrates the radiative thermal load profile of Non‐PL films with structural or pigmentary color generated only by intrinsic absorption. In this scenario, the thermal load ranges from ‐120 to 340 W m^−2^ with the target chromaticity coordinate (0.3728, 0.3029) exhibiting a thermal load of ‐47 W/m^2^. When considering the PL process, as typically observed in planar polymeric films such as PMMA or cellulose with refractive indices ≈1.5, ≈75% of the re‐emitted light becomes trapped within the film, contributing to heat generation. Without LE efficiency enhancement, only 25% of light can be extracted (Supporting Information Section 2). Figure 2c depicts the thermal load distribution ranging from ‐122 to 308 W m^−2^ without the improvement of LE. The thermal load at the target chromaticity coordinate (0.3728, 0.3029) is −58 W m^−2^. Figure 2d represents the scenario where LE efficiency is idealized at its theoretical maximum of 100% (neglecting self‐absorption effects), wherein energy losses arise solely from internal quantum yield and down‐shifting processes. Under these conditions, the thermal load varies from ‐126 to 140 W m^−2^. The thermal load at the target chromaticity coordinate (0.3728, 0.3029) is −98 W m^−2^.

These three thermal distribution surfaces feature white boundary lines representing zero thermal load. Above these boundaries, red regions indicate positive thermal loads (above‐ambient), while teal regions below denote negative thermal loads (sub‐ambient). As LE efficiency increases, the surface shifts toward decreased power values, with expanding white boundaries and significant downward movement of the upper surface edge. This indicates overall thermal load reduction and expansion of the sub‐ambient color range. Figure 2e displays the projections of the white critical boundary lines onto the xy chromaticity plane under Non‐PL, 25% LE, and 100% LE scenarios, represented by red, gray, and blue dash lines, respectively. The proportional areas enclosed by these boundaries within the CIE 1931 color space are 19.3%, 26.7%, and 64.9%, respectively.

### 2.3 Ray‐Tracing Simulation of Scattering Film

**Figure 3:**
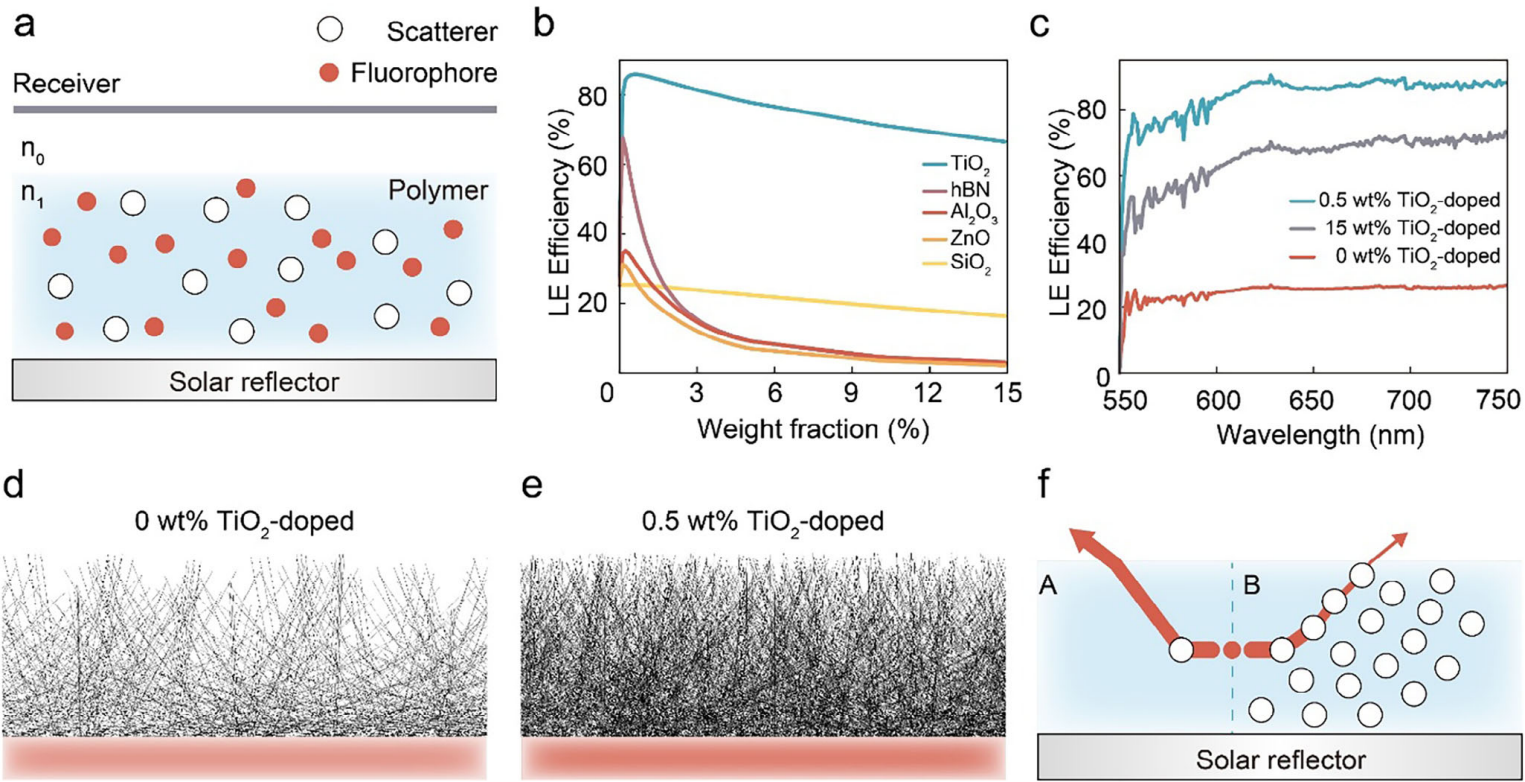
LE efficiency in fluorescent films is influenced by different scattering agents and doping concentration ​. a) Side‐view schematic of a fluorophore‐doped PMMA film (*n*_1_​ = 1.5) with embedded spherical scatterers and fluorophores. A specular solar reflector is placed at the bottom. Air (*n*_0_​ = 1.0) surrounds the film. b) LE efficiency versus scatterer weight fraction. TiO_2_ achieves peak LE (85.9% at 0.5 wt%) due to its high refractive index contrast with PMMA and low extinction coefficient (*k*), maximizing scattering and minimizing absorption. c) LE efficiency spectra for undoped (0 wt%, 25.3%), optimally doped (0.5 wt% TiO_2_, 85.9%), and overdoped (15 wt% TiO_2_, 66.6%) films. Simulated ray trajectories are illustrated in d) undoped and e) optimal 0.5 wt% TiO_2_‐doped films. Controlled scattering in the doped film redirects trapped photons toward the escape cone, enhancing LE. f) At low doping (Region A), moderate scattering preserves re‐emitted light intensity while redirecting paths. At high doping (Region B), excessive scattering events attenuate the re‐emitted light intensity, resulting in low LE efficiency.

**Figure** **3**a presents a side view of the fluorophore‐doped films containing scattering particles. The polymer film (refractive index *n*_1_) contains uniformly distributed fluorophores and scatterers, with its bottom surface adjacent to a highly specular solar reflector. The polymer film is surrounded by air (refractive index *n*_0_). For practical simulation, the homogeneous distribution of fluorophores within the polymer matrix can be modeled as an embedded volumetric source. The volume source with dimensions slightly smaller than the host polymer preserves the identical optical properties of the polymer material. We selected PMMA (*n* = 1.5) as the host matrix due to its high visible light transparency and employed the emission spectrum of commercial fluorescent material Lumogen F Red 305 (LF305, BASF) with its high quantum yield (≈90%) (Supporting Information Section 3). When investigating the relationship between different scattering agents and LE efficiency, we assumed spherical scatterers to eliminate geometric variables and verified the influence of intrinsic optical properties, specifically refractive index and extinction coefficient. Figure 3b presents LE efficiency curves for various scattering agents^[^ ^38^, ^39^, ^40^, ^41^, ^42^ ^]^ used to enhance solar reflectance, plotted as functions of weight fraction. Except for SiO_2_, the LE curves exhibit similar trends: initially increasing dramatically to maximum values, then decreasing and converging, though with significantly varying rates of change. For SiO_2_, due to its refractive index in the visible light band of 550–750nm, which is the fluorophore emissive wavelength band, approaching that of the matrix material (Figure S2b, Supporting Information), scattering effects are minimal. Comparing the refractive index (*n*) and extinction coefficient (*k*) of these scatterers with their corresponding LE efficiency curves reveals that high *n* and low *k* are essential determinants of a film's LE capacity. For internally re‐emitted light, greater refractive index differential between matrix and scatterer enhances scattering effects, while low extinction coefficients minimize absorption losses during increased scattering events, preserving emission intensity while altering propagation paths. Therefore, TiO_2_, possessing the highest refractive index and lowest extinction coefficient among the evaluated scatterers (Figure S2b, Supporting Information), demonstrated superior LE performance. At 0.5 wt% doping concentration, it achieved peak LE efficiency of 85.9%, with the detailed spectral distribution shown in Figure 3c, which also displays LE efficiency spectra under two reference scenarios: without scatterers (0 wt% TiO_2_ doping) and with high concentration doping (15 wt% TiO_2_ doping), yielding total LE efficiencies of 25.3% and 66.6%, respectively. Figure 3d,e provide a comparative visualization of simulated ray trajectories, demonstrating the marked difference in light extraction capabilities between optimally doped and undoped control samples.

For fluorescent films, LE efficiency directly correlates with effective solar reflectance. The significant differential in LE efficiency between optimal (0.5 wt%) and high (15 wt%) doping concentrations (85.9% vs 66.6%) indicates that moderately doped films can potentially achieve higher effective solar reflectance despite their lower scatterer concentration. This result is counterintuitive, as films with higher scatterer concentrations typically exhibit higher surface reflectance. However, the intensity of the reflected light caused by scattering at the surface will compete with the absorption intensity of the fluorophore within the film. Moreover, as illustrated in Figure 3f, the light exhibits different scattering behavior in the low‐doping concentration region (A) versus the high‐doping concentration region (B). If the scattering concentration becomes excessively high, the intensity of the internally re‐emitted light will dramatically decrease after undergoing a large number of scattering events. Conversely, an appropriate amount of scatterers can redirect the light's propagation direction while preserving the re‐emitted light intensity, thereby extracting more light outside the film. Therefore, based on the simulation result of LE efficiency of the low and high TiO_2_ concentration doping, a fluorescent film with lower lightness coloration but with better reflectance is possible.

### 2.4 Optical Characterization

**Figure 4:**
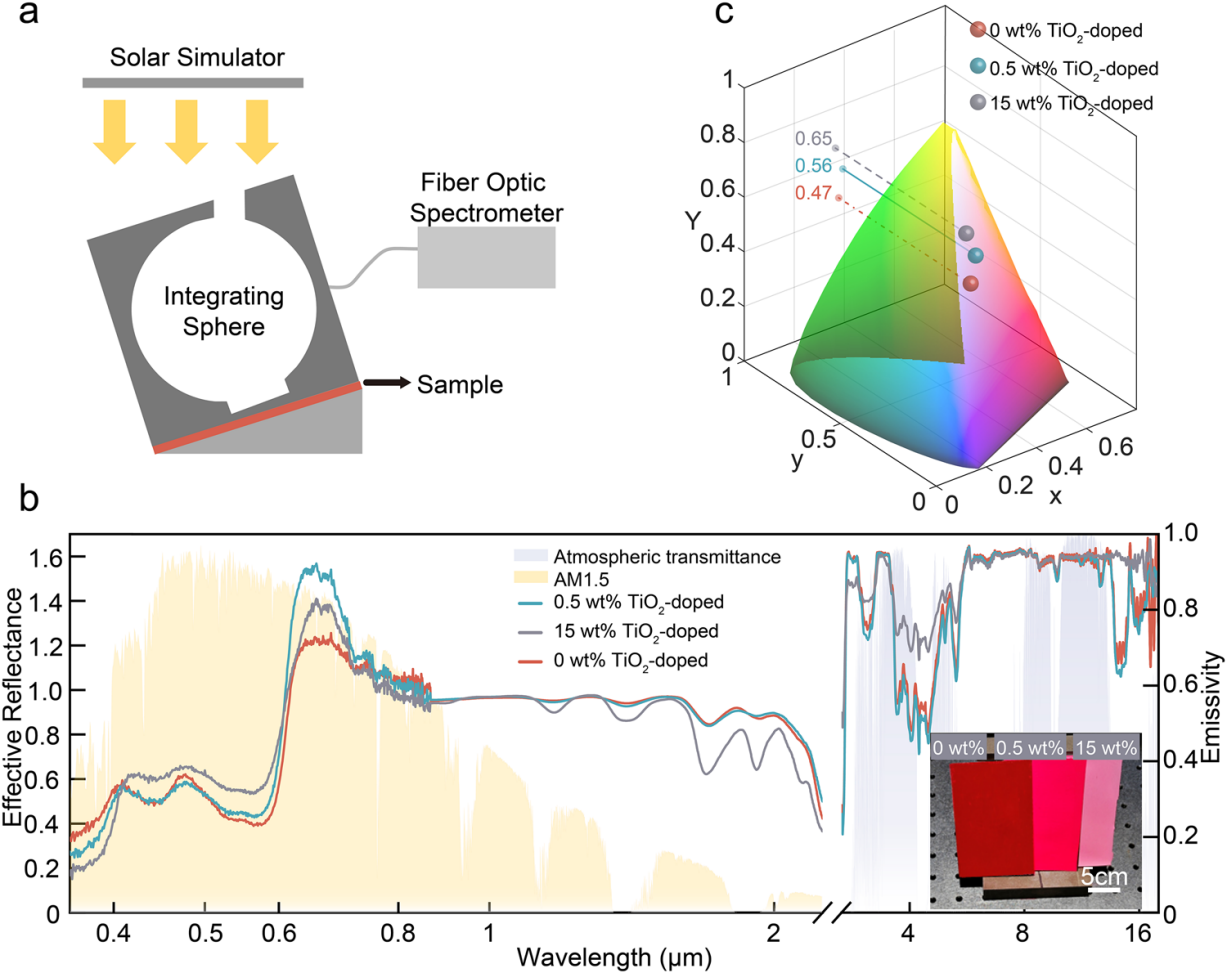
LE optimization enhances solar reflectance despite lower lightness in fluorescent films. a) Reflectance measurement setup using an integrating sphere of 50 mm diameter under solar simulator illumination. Films are angled at 8° to maintain collimated light. Reflected and re‐emitted light is collected via a fiber spectrometer. b) Effective solar reflectance (0.36–2.5 µm) and infrared emissivity for films with 0 wt%, 0.5 wt%, and 15 wt% TiO_2_. The 0.5 wt% sample achieves the highest total solar reflectance (89.5%) and lowest absorbed solar power (105 W m^−2^), despite the lower TiO_2_ concentration than the 15 wt% sample (86.2% reflectance, 138 W m^−2^ absorbed solar power). The reflectance of the control undoped sample is 83.1%, absorbing 169 W m^−2^ of solar power. The inset shows the film photograph (left to right: 0, 0.5, 15 wt%). c) CIE Yxy color coordinates. Chromaticity (x,y) overlaps across samples, but lightness (Y) increases with growing TiO_2_ concentration (47%, 56%, 65%). Counterintuitively, the 0.5 wt.% sample (moderate lightness) achieves the highest solar reflectance than the 15 wt% sample (high lightness).

Guided by the simulation results, we prepared three fluorescent‐colored samples with TiO_2_ nanoparticle loadings of 0 wt% (control), 0.5 wt%, and 15 wt%. The commercial spectrometer operates on the principle of measuring reflectivity at the same wavelength as the light source. However, this methodology fails to account for the spectrum of the fluorescent film, which re‐emits light at a different wavelength from the source light. Therefore, we employed a custom setup to measure the reflectance spectrum of the fluorescent film, as illustrated in **Figure** **4**a. An integrating sphere with a diameter of 50 mm (BASI Optoelectronic) was positioned under a solar simulator (94083A, Newport). The sphere features a port positioned at 8 degrees for direct illumination. Both the film and the integrating sphere were positioned on an 8‐degree angled slope to maintain collimated light. Reflected and re‐emitted light was collected using a fiber spectrometer (S2000, Ocean Optics). The measured effective solar reflectance and infrared emissivity of these samples are presented in Figure 4b, with the inset showing photographs of the samples from left to right with increasing TiO_2_ concentration. In addition to the characterization of the visible spectrum, infrared spectrum characterization is introduced in the Materials and Methods section. The low‐concentration TiO_2_‐doped and undoped samples exhibit comparable LF305‐induced absorption profiles in the visible spectrum, while a marked difference of ≈40% in their reflective peak values within the fluorophore emission band. For the high concentration TiO_2_‐doped sample, substantial TiO_2_ scattering enhances the solar reflection at the surface of the sample, reducing the absorbed energy by the fluorophores and the emitted light intensity, consequently. In addition, when the TiO2 concentration exceeds the optimal doping concentration, the light extraction efficiency will decrease. Although the LE efficiency of the high‐concentration TiO_2_‐doped sample reaches 66.6%, its reflective peak value remains ≈20% lower than that of the low‐concentration TiO_2_‐doped sample. Overall, the total visible reflectance for undoped, low‐concentration‐doped, and high‐concentration‐doped samples are 78.5%, 86.1%, and 84.0%, respectively, and their corresponding total solar reflectance values are 83.1%, 89.5%, and 86.2%. Consequently, under standardized solar irradiance (1000 W m^−2^), the solar absorbed power for the undoped, low‐concentration‐doped, and high‐concentration‐doped samples are 169, 105, and 138 W m^−2^, respectively. The net thermal radiation power is almost consistent across all samples at ≈112 W m^−2^ at 300K. The detailed power balance analysis is provided in Supporting Information Section 1. The inset in Figure 4b directly displays the color appearance of three samples, with TiO_2_ doping concentrations of 0, 0.5, and 15 wt% from left to right, respectively. Counterintuitively, although the 15 wt% doped sample with the lightest color, it has a higher radiative thermal load than the optimal 0.5 wt% doped sample.

Based on the visible reflectance spectra, we calculated the color coordinates shown in Figure 4c. While the chromaticity of all three samples is essentially identical, resulting in an overlap within the CIE 1931 color space, their lightness values differ. To demonstrate these lightness variations, we mapped their color coordinates to the CIE Yxy color space, where Y represents lightness and coordinates x and y determine chromaticity.^[^ ^43^, ^44^ ^]^ The lightness (Y) values for these samples are 47%, 56%, and 65%, corresponding to TiO_2_ concentrations of 0, 0.5, and 15 wt%, respectively. Thus, by doping optimal scatterer concentration (0.5 wt%) to enhance LE efficiency in fluorescent films, we achieved high solar reflectance compared to that without LE improvement. In addition, modulating concentration to regulate LE efficiency realized the counterintuitive result wherein the film with lower lightness coloration (0.5 wt% sample) exhibited higher effective solar reflectance than those with higher lightness coloration (15 wt% sample).

### 2.5 Outdoor Experiment Result

**Figure 5:**
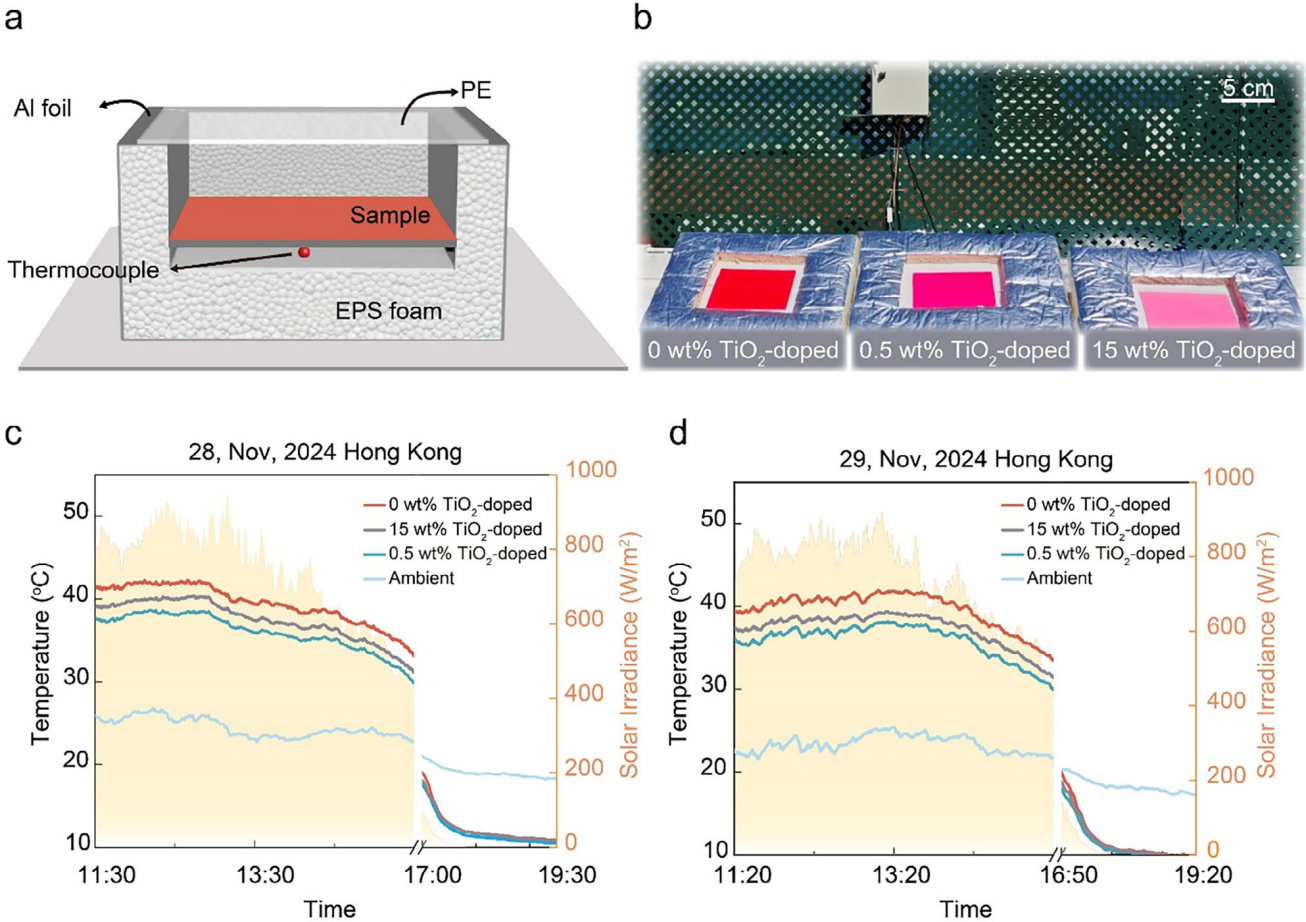
Outdoor testing of fluorescent samples with varying TiO_2_ concentrations. a) Schematic diagram of the experimental setup consisting of three identical chambers positioned side by side, each containing a different sample, with a thermocouple attached to the bottom of the sample for temperature measurement and a polyethylene film covering to allow radiative heat exchange while minimizing convective and conductive heat transfer. b) Photograph of the actual experimental setup deployed outdoors. c) and d) Temperature variation curves for the three samples (0 wt%, 0.5 wt%, and 15 wt% TiO_2_) and ambient conditions measured on continuous two days, November 28 and 29, 2024, showing significant daytime temperature differences between samples while maintaining equivalent nighttime radiative cooling performance. Daytime temperature differences arise from solar reflectance variations. The optimally doped sample (0.5 wt.% TiO_2_) demonstrated the lowest daytime temperature, averaging 3.8 °C cooler than the undoped sample and 1.5 °C cooler than the highly doped sample during peak solar hours on November 28, 2024. A similar temperature trend was also observed on November 29, 2024.

We characterized the thermal performance of the three samples on a building rooftop during clear‐sky days in Hong Kong, China. **Figure** **5**a,b present a diagram and photograph of the experimental setup, comprising three identical chambers placed adjacently, each housing a different sample. Each chamber was constructed from high‐density expanded polystyrene (EPS) foam covered with aluminum film for thermal insulation. To minimize non‐radiative heat transfer between samples and the surroundings while enabling radiative exchange between the samples and external radiation sources (sun, atmosphere, and outer space), we covered the chamber openings with 10‐µm‐thick polyethylene film, a material transparent to solar and infrared radiation. Sample temperatures were measured by thermocouples positioned at the interface between each sample and its supporting foam. A calibrated commercial weather station equipped with a louvered enclosure was used to record ambient temperature, relative humidity, and solar irradiance.

Figure 5c,d present temperature variation curves for the three samples, primarily capturing the period of maximum solar irradiance on 28–29 November 2024. On November 28, temperature profiles of all three samples converged at night to ≈10.5 °C, which was 7.8 °C below the ambient temperature of 18.3 °C, confirming that daytime temperature differentials between the fluorescent samples primarily result from variations in effective solar reflectance, as their radiative cooling performances are equivalent. During the primary 4‐h daytime measurement interval, the average temperature difference between samples doped with 0 and 0.5 wt% TiO_2_ was 3.8 °C, with a maximum differential of 4.1 °C observed at noon when solar irradiance exceeded 850 W/m^2^. The average temperature difference between samples doped with 15 and 0.5 wt% TiO_2_ was 1.5 °C, with a maximum differential of 2.0 °C. Temperature profiles on November 29 showed consistent performance with the previous day's temperature measurement, with all samples converging to ≈10 °C at night (7.5 °C below ambient temperature) while exhibiting maximum 4.1 °C daytime temperature differences between 0 wt% and 0.5 wt% samples, also maximum 1.9 °C between 15 wt% and 0.5 wt% samples. Additional experimental results of humidity are presented in Figure S7a,b (Supporting Information). Figure S8 (Supporting Information) presents the temperature records of low‐concentration TiO_2_‐doped and undoped fluorescent films in summer, with the reduced average temperature difference likely attributed to higher ambient temperature and humidity in summer.

## 3 Conclusion

Our study has demonstrated that enhanced light extraction, achieved through multiple scattering events induced by nanoparticles, substantially reduces the radiative thermal loads of fluorescent film. Our optimization model, which incorporates the light‐extraction coupled down‐shifting process, indicates a significant decrease in thermal load facilitated by enhanced light extraction efficiency from 25% to 100%, which expands the sub‐ambient cooling color range from 26.7% to 64.9% within the CIE 1931 space. An optimal 0.5 wt% TiO_2_ doping concentration boosted the light extraction efficiency to 85.9% and total solar reflectance to 89.5%. Outdoor testing was validated with the 0.5 wt% sample, demonstrating a 4.1 °C temperature reduction compared to the undoped film during peak irradiance. Our findings indicate that regulating the light extraction efficiency of fluorescent films offers a promising approach to resolving the traditional trade‐off between color display range and cooling performance, expanding the potential applications of energy‐saving technologies with vibrant coloration in outdoor environments.

## 4 Experimental Section

### Fabrication and Characterizations

The scattering‐enhanced light‐extracting films were fabricated using a blade coating method, as illustrated in Figure S3 (Supporting Information). The resulting films were trimmed into rectangular samples with dimensions of 10 cm × 7 cm × 0.015 cm for experimental measurement. Fluorescent polymer solutions containing TiO_2_ (anatase, 100–300 nm particle size, Macklin) were prepared by dissolving LF305 and commercial‐grade poly(methyl methacrylate) (PMMA) (180000 molecular weight, Aladdin Scientific) in N,N‐dimethylformamide (DMF) (Aladdin Scientific). Solutions were mixed at ambient temperature for 24 h with continuous magnetic stirring. The overall solute concentration was standardized at 15 g per 100 mL of DMF solvent. LF305 was maintained at a fixed concentration of 0.1 wt% of the total solute mass (0.015 g per 15 g total solute). TiO_2_ nanoparticles were incorporated at three distinct mass fractions: 0 wt% (control), 0.5 wt%, and 15 wt%. PMMA served as the matrix material, with its mass fraction adjusted inversely to TiO_2_ content to maintain a constant total solute mass of 15 g. For example, in the 15 wt% TiO_2_ formulation, PMMA constituted 84.9 wt% of the solute.

An automated blade coating film applicator (MSK‐AFA‐II, MTI) and an adjustable blade were employed to produce large‐area films with controlled wet thicknesses. During film preparation, a glass plate served as the substrate for blade coating. The entire apparatus was enclosed within an acrylic chamber, and after coating completion, dry compressed gas was continuously introduced to maintain a low‐humidity environment, preventing DMF solvent from absorbing ambient moisture before complete evaporation, which would otherwise result in the formation of porous, white opaque PMMA films.

The spectral characteristics of the scattering‐enhanced light‐extracting films, with silver‐plated aluminum plates as substrates, were analyzed using various instrumental techniques. The reflectance spectrum in the near‐infrared region (0.86 to 2.5 µm) was measured by UV–Vis‐NIR spectrophotometer (Cary 5000, Agilent) with a 110 mm integrating sphere (DRA‐2500, Agilent). The emissivity spectrum in the infrared region (2.5 to 20 µm) was measured using a Fourier transform infrared spectrometer (FT‐IR) (Vertex, Bruker) with a gold‐coated integrating sphere (PIKE Technologies).

### Ray‐Tracing Simulation Setup

The geometric dimensions of the film are 10 × 10 × 0.15 mm^3^, with a receiver positioned adjacent to the top surface at a fixed gap distance of 1 µm. The bottom surface exhibits optical properties of 95% reflection and 5% absorption, while the optical properties of all other surfaces are determined using the Fresnel equations. The scatterers have a diameter of 200 nm, and the light extraction efficiency for different scattering materials was calculated using their respective refractive indices and extinction coefficients, as shown in Figure S2b,c (Supporting Information). To obtain accurate light extraction efficiency values, we employed Monte Carlo‐based ray‐tracing methods, which utilize both probability theory and mathematical statistics. One million randomly generated rays were traced to ensure computational accuracy, with a relative ray power threshold of 10^−3^, meaning that ray tracing terminates when a light ray's energy decays to 10^−3^ of its original value.

## Conflict of Interest

The authors declare no conflict of interest.

## Supporting information



Supporting Information

## Data Availability

The data that support the findings of this study are available from the corresponding author upon reasonable request.
