# Supplementary material for: Scattering‐Enhanced Light Extraction for Radiative Thermal Load Mitigation in Fluorescent Films
Source: Adv Sci (Weinh). 2025 Sep 15;12(43):e10643. doi: 10.1002/advs.202510643 (PMC12631933; doi:10.1002/advs.202510643)
Supplement: Supplementary file 1 — Supporting Information [file ADVS-12-e10643-s001.docx]

**Supporting Information for**

Scattering-enhanced light extraction for radiative thermal load mitigation in fluorescent films

*Chenglong She* ^1 †^, *Yi Zhang* ^1†^, *Minghao Dong* ^1^, *Xiaopeng Bai* ^1^, *Chenxi Wang* ^1^, *Fan Yang* ^1^, and *Xiaobo Yin* ^1, 2, *^

^1^Department of Mechanical Engineering, The University of Hong Kong, Hong Kong 999077, China

^2^Department of Physics, The University of Hong Kong, Hong Kong 999077, China

^†^ C.S and Y.Z contributed equally to this work

^*^ Corresponding E-mail: xbyin@hku.hk

Section 1 Optimal radiative thermal load calculation

1.1 Radiative heat exchange

The total radiative thermal load $P_{thermal}$ (W/m^2^) can be expressed as:

$$\begin{aligned} P_{thermal}=P_{UV}+P_{VIS}+P_{NIR}-(P_{rad}-P_{atm})\#\left( 1 \right) \end{aligned}$$

The absorbed solar power is partitioned into ultraviolet, visible and near-infrared absorption components, $P_{UV}$,$P_{VIS}$ and $P_{NIR}$:

$$\begin{aligned} P_{UV}=\int_{280 nm}^{360 nm} I_{AM1.5}\left( \lambda\right)\cdot\left[ 1-r\left( \lambda\right) \right]d\lambda\#\left( 2 \right) \end{aligned}$$

$$\begin{aligned} P_{VIS}=\int_{360 nm}^{760 nm} I_{AM1.5}\left( \lambda\right)\cdot\left[ 1-r\left( \lambda\right) \right]d\lambda\#\left( 3 \right) \end{aligned}$$

$$\begin{aligned} P_{NIR}=\int_{760 nm}^{4000 nm} I_{AM1.5}\left( \lambda\right)\cdot\left[ 1-r\left( \lambda\right) \right]d\lambda\#\left( 4 \right) \end{aligned}$$

Where $r\left( \lambda\right)$ represents the wavelength-dependent reflectance spectrum, with $1-r\left( \lambda\right)$ denoting the absorptivity spectrum. $I_{AM1.5}\left( \lambda\right)$ signifies the solar irradiance according to the AM1.5 spectrum (W/m^2^/nm). The term $P_{rad}-P_{atm}$ is the net thermal radiation power:

$$\begin{aligned} P_{rad}=\int\left( \int I_{BB}\left( \lambda, T \right)\cdot\varepsilon\left( \lambda\right) \right)\cdot\cos\theta d\omega\#\left( 5 \right) \end{aligned}$$

$P_{rad}$ denotes the emissive power of objects after hemispherical integration. $I_{BB}\left( \lambda, T_{atm} \right)=\left( \frac{2hc^{2}}{\lambda^{5}} \right)\cdot\left( e^{\frac{hc}{\lambda k_{B}T}}-1 \right)^{-1}$ represents the spectral intensity of blackbody radiation. $\varepsilon\left( \lambda\right)$ is the absorptivity of objects, which equals the emissivity. Assuming the objects are isotropically emissive and opaque, $\varepsilon\left( \lambda\right)$ is angle-independent and equals $1-r\left( \lambda\right)$. The hemispherical integration $\Omega=\int\cos\theta d\omega=\int_{0}^{\pi/2} \sin\theta\cos\theta d\theta\int_{0}^{2\pi} d\phi$, where $\theta$ and $\phi$ denote the zenith and azimuth angle respectively.

$$\begin{aligned} P_{atm}=\int\left( \int I_{BB}\left( \lambda, T_{atm} \right)\cdot\varepsilon_{atm}(\lambda, \theta)\cdot\varepsilon(\lambda)d\lambda\right)\cdot\cos\theta d\omega\#\left( 6 \right) \end{aligned}$$

$P_{atm}$ represents the power absorbed from atmospheric radiation after hemispherical integration. $\varepsilon_{atm}\left( \lambda, \theta\right)=1-{t(\lambda)}^{1/\cos\theta}$ denotes the emittance of the atmosphere, which depends on the zenith angle $\theta$. This can be obtained by Beer-Lambert law, which assumes the transmittance along the vertical direction is $t=e^{(-\tau)}$, and $\tau$ is optical thickness indicating how strongly a medium attenuates radiation at a given wavelength. Therefore, when radiation passes through the atmosphere at a zenith angle $\theta$, their actual path length is $1/cos\theta$ times the vertical path, $t\left( \theta\right)=e^{-\tau/cos\theta}={(e^{-\tau})}^{1/cos\theta}=t^{1/cos\theta}$.

1.2 Visible reflectance to color

The CIE 1931 chromaticity coordinate ($x,y$) are derived from the tristimulus values *X*, *Y*, and *Z*, which quantify the human visual system's response to spectral stimulus under a defined illuminant. For a visible reflectance spectrum $r\left( \lambda\right)$, the tristimulus values are computed as:^1,2^

$$\begin{aligned} X=k\int_{360 nm}^{760 nm} I\left( \lambda\right)\cdot r\left( \lambda\right)\cdot\bar{x}\left( \lambda\right)d\lambda\#\left( 7 \right) \end{aligned}$$

$$\begin{aligned} Y=k\int_{360 nm}^{760 nm} I\left( \lambda\right)\cdot r\left( \lambda\right)\cdot\bar{y}\left( \lambda\right)d\lambda\#\left( 8 \right) \end{aligned}$$

$$\begin{aligned} Z=k\int_{360 nm}^{760 nm} I\left( \lambda\right)\cdot r\left( \lambda\right)\cdot\bar{z}\left( \lambda\right)d\lambda\#\left( 9 \right) \end{aligned}$$

$$\begin{aligned} k=\frac{100}{\int_{360 nm}^{760 nm} I\left( \lambda\right)\cdot\bar{y}\left( \lambda\right)d\lambda}\#\left( 10 \right) \end{aligned}$$

where $I\left( \lambda\right)$ represents the spectral power distribution of the illuminant (e.g., D65 standard illuminant). $\bar{x}\left( \lambda\right)$, $\bar{y}\left( \lambda\right)$ and $\bar{z}\left( \lambda\right)$ are CIE 1931 color-matching functions (CMFs), representing the spectral sensitivity of human cone cells. The constant *k* ensures that a perfectly reflective surface ($r\left( \lambda\right)=1$ for all $\lambda$) yields $Y=100$, conforming to CIE standards. The chromaticity coordinates ($x,y$) are obtained by normalizing the tristimulus values:

$$\begin{aligned} x=\frac{X}{X+Y+Z}\#\left( 11 \right) \end{aligned}$$

$$\begin{aligned} y=\frac{Y}{X+Y+Z}\#\left( 12 \right) \end{aligned}$$

These coordinates project the tristimulus values onto the CIE 1931 *xy* chromaticity diagram, effectively decoupling color information from luminance *Y*.

1.3 Fluorescence modeling

Photoluminescence (PL) in this work redistributes absorbed energy into longer wavelengths. The emission spectrum $E\left( \lambda\right)$ is modeled as a normal distribution:

$$\begin{aligned} E\left( \lambda\right)=\frac{P_{conversion}}{\sigma\sqrt{2\pi}}\cdot\exp\left( -\frac{\left( \lambda-\mu\right)^{2}}{2\sigma^{2}} \right)\#\left( 13 \right) \end{aligned}$$

$$\begin{aligned} P_{conversion}=\eta_{ext}\cdot QY\cdot SS\cdot\int_{360}^{\lambda_{0}} I_{AM1.5}\left( \lambda\right)\cdot\left[ 1-r\left( \lambda\right) \right]d\lambda\#\left( 14 \right) \end{aligned}$$

where $\lambda_{0}$ represents the absorption cut-off wavelength. The mean wavelength $\mu=\frac{\lambda_{0}+760}{2}$ ensures the emission peak is centered between $\lambda_{0}$ and 760 nm, while the standard deviation $\sigma=\frac{760-\lambda_{0}}{4}$ confines 95% of emission within $\lambda_{0}$ and 760 nm ($\pm2\sigma$). $P_{conversion}$ quantifies the power converted via PL, with $\eta_{ext}$ (​light extraction efficiency) ranging from 25% (planar surface, refractive index 1.5) to 100% (ideal extraction), $QY$ (quantum yield) and $SS=\frac{360+\lambda_{0}}{760+\lambda_{0}}$ (stokes shift). The visible reflectance $r\left( \lambda\right)$ is augmented as $r\left( \lambda\right)+\frac{E\left( \lambda\right)}{I_{AM1.5}}$ for $\lambda_{0}\leq\lambda\leq760$nm, accounting for re-emitted photons escaping the material. For exploring the theoretical limit, we ignored energy losses from self-absorption processes.

1.4 Optimization algorithm

The Sequential Least Squares Quadratic Programming (SLSQP) algorithm was employed to solve the constrained nonlinear optimization problem defined in Equation set (6) due to its compatibility with nonlinear constraints, bounds on variables, and high-dimensional parameter spaces.^3^ SLSQP efficiently handles the chromaticity constraint ($\Delta E\leq\Delta$) and reflectance bounds $0\leq r\left( \lambda\right)\leq1$ by iteratively approximating the problem as quadratic programming (QP) subproblems. Its gradient-based updates and ability to leverage quasi-Newton Hessian approximations ensure robust convergence in non-convex, multidisciplinary spectral optimization tasks.^4^

The original optimization problem involves minimizing the objective function $P_{VIS}$ subjected to chromaticity constraints and reflectance bounds. To integrate these constraints, the Lagrangian function $\mathcal{L}$ is formulated as:

$$\begin{aligned} \mathcal{L}\left( r, \mu\right)=P_{VIS}+\mu_{\Delta E}\left( \Delta E-\Delta\right)+\sum_{\lambda} \left[ \mu_{lower,\lambda}\left( -r\left( \lambda\right) \right)+\mu_{upper,\lambda}\left( r\left( \lambda\right)-1 \right) \right]\#\left( 15 \right) \end{aligned}$$

where $\mu$ is Lagrange multiplier set. Here it includes$\mu_{\Delta E}$, $\mu_{lower,\lambda}$, and $\mu_{upper,\lambda}$. The chromaticity constraint is a ​global constraint involving all wavelengths, hence $\mu_{\Delta E}$ is a scalar. Reflectance bounds are ​local constraints depending on each wavelength, resulting in 2N multipliers ($\mu_{lower,\lambda}, \mu_{upper,\lambda}$). *N* means that spectral optimization problem with *N* discretized wavelengths.

**Objective simplification**: Once the initial value of the reflectance $r$ is given, at the following iteration *k*, the Lagrangian function $\mathcal{L}$ with value $r_{k}$ is approximated quadratically. Adopting Taylor second-order expansion, the quadratic approximation retains the first-order gradient of the objective function $P_{VIS}$ and second-order Hessian terms of the Lagrangian function $\mathcal{L}$ at $r_{k}$:

$$\begin{aligned} \mathcal{L}\left( r_{k}(\lambda)+\Delta r^{k}(\lambda) \right)\approx\nabla P_{VIS}^{k}\cdot\Delta r^{k}(\lambda)+\frac{1}{2}\cdot{\mathbf{H}_{k}\cdot\left( \Delta r^{k}(\lambda) \right)}^{2}\#\left( 16 \right) \end{aligned}$$

where $\Delta r^{k}(\lambda)$ represents optimal step as the variable to be optimized in iteration *k*, $\nabla P_{VIS}^{k}\cdot\Delta r^{k}(\lambda)=\int\frac{\delta P_{VIS}}{\delta r\left( \lambda\right)}\Delta r^{k}(\lambda)d\lambda=-\int I_{AM1.5}\left( \lambda\right)\Delta r^{k}(\lambda)d\lambda$ denoted as the first-order term of functional Taylor expansion, and $\frac{\delta P_{VIS}}{\delta r\left( \lambda\right)}$ is functional derivative. $\mathbf{H}_{k}$ is the Hessian matrix approximated via quasi-Newton methods (e.g., BFGS). The Lagrange multiplier is included in it.

**Constraint linearization**: nonlinear constraints are expanded to first order using Taylor series:

$$\begin{aligned} \Delta E\approx{\Delta E}_{k}+\sum_{\lambda} \frac{\partial\Delta E}{\partial r\left( \lambda\right)}\Delta r^{k}(\lambda)\leq\Delta\#\left( 17 \right) \end{aligned}$$

where $\frac{\partial\Delta E}{\partial r\left( \lambda\right)}$ is derived from the chain rule applied to the CIE tristimulus values, incorporating gradients of tristimulus values (e.g., $\frac{\delta X}{\delta r\left( \lambda\right)}=kI(\lambda)\bar{x}\left( \lambda\right)$, $X$ is also a functional of $r\left( \lambda\right)$). Reflectance bounds are directly enforced as linear inequalities.

**Implementation process:**

1**.** Gradient Computation: $\nabla P_{VIS}\cdot\Delta r(\lambda)$ and $\frac{\partial\Delta E}{\partial r\left( \lambda\right)}$ are computed analytically.

2. Hessian Approximation: Updated iteratively using the BFGS method to capture curvature without explicit second-derivative calculations.

3. Constraint Linearization: Chromaticity constraints are linearized at each iteration.

4. QP Subproblem Solution: A convex QP solver determines the optimal step $\Delta r(\lambda)$.

5. Step Size Adaptation: A line search ensures sufficient decrease in $P_{VIS}$​ while satisfying constraints.

If considering the PL process, the cut-off wavelength $\lambda_{0}$ should be added to the variable set to participate in the optimization loop and normal distribution process also needs to be considered in the objective function and constraint function.

Section 2 Limited light extraction efficiency of the planar fluorescent films

Figure S1 illustrates a fluorescent particle emitting light isotropically within a polymer medium (refractive index $n_{1}$) into a medium with (refractive index $n_{0}$). The polymer features flat top and bottom surfaces. When $n_{1}>n_{0}$, a significant portion of emitted light becomes trapped within the film due to total internal reflection (TIR). The red dash line represents the escape cone Ω, which depicts the probability of photons escaping from the thin film, corresponding to twice the critical angle $\theta_{c}=\sin^{-1} (\frac{n_{0}}{n_{1}})$. The solid red arrows within Ω represent rays that can escape the polymer, while solid black arrows outside Ω represent rays trapped within the polymer. Light extraction efficiency is therefore limited by the dimensions of this escape cone.

In three-dimensional space, the escape angle is characterized by the solid angle shown on the right side of Figure S1. The solid angle of escape cone Ω $= \int_{0}^{\theta_{c}} \sin\theta d\theta\int_{0}^{2\pi} d\varphi=2\pi(1-\cos\theta_{c})$ represents the integral of the zenith angle from 0 to $\theta_{c}$ and azimuth from 0 to $2\pi$. For isotropic emission, due to the vertical geometric symmetry of the medium, the light extraction efficiency equals the ratio of the solid angle of the escape cone Ω to the hemisphere solid angle $2\pi$, i.e., $\eta_{extraction}= \frac{}{2\pi}=(1-\cos\theta_{c})$. Therefore, when $n_{0}=1$ and $n_{1}=1.5$ (typical averaged refractive indices of air and polymers for visible light), the light extraction efficiency for fluorophore emission from polymer to air is approximately 25%, with nearly 75% of light remaining trapped within the polymer, undergoing multiple reflections at the top and bottom planar surfaces.^5^


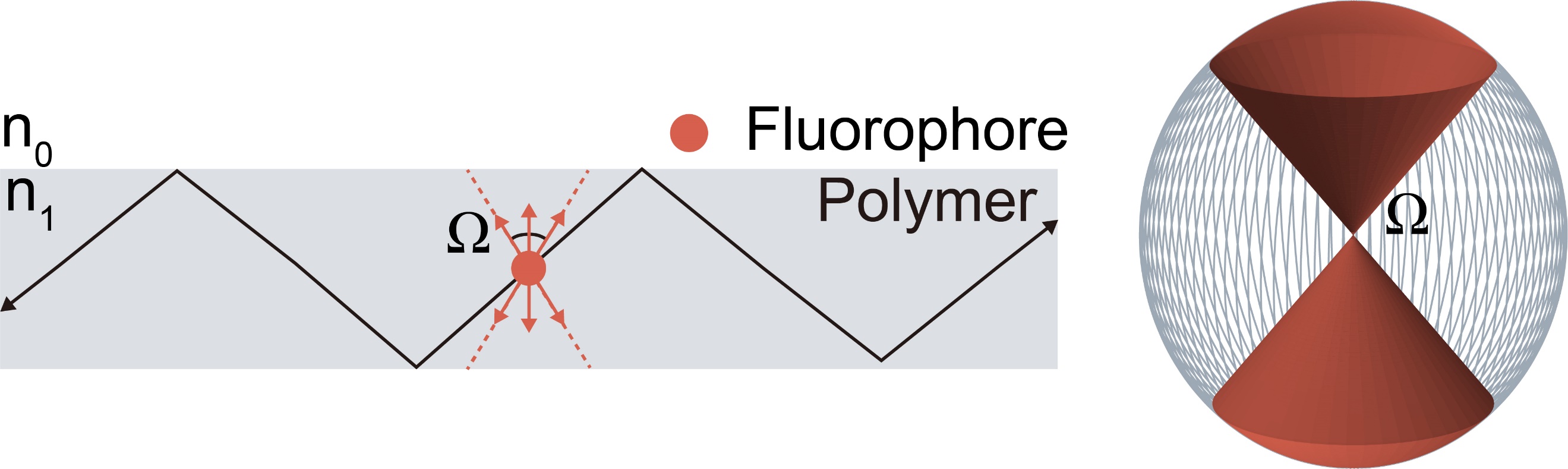


**Figure S1. Theoretical limitations of light extraction from planar fluorescent films due to TIR.** Schematic representation of light emission from a fluorescent particle embedded in a polymer medium (refractive index $n_{1}$) into surrounding medium (refractive index $n_{0}$), showing the escape cone Ω (red dashed line) defined by the critical angle $\theta_{c}=\sin^{-1} (n_{0}/n_{1})$. Rays within the escaped cone (red arrows) can exit the polymer, while rays outside this cone (black arrows) remain trapped due to TIR. The light extraction efficiency equals $(1-\cos\theta_{c})$, resulting in approximately 25% extraction efficiency for typical polymer-air interfaces ($n_{1}$=1.5, $n_{0}$=1), with 75% of light remaining trapped within the polymer layer.

Section 3 Spectra for simulation of light extraction efficiency

The emission energy distribution of each emissive volume element inside the film corresponds to the emission spectrum of commercial fluorescent powder Lumogen F Red 305 (LF305, BASF), as shown in Figure S2a. Consequently, light extraction efficiency equals the ratio of total received energy in the reflected light side to the total emitted energy, disregarding losses from self-absorption effects. To calculate the light extraction efficiency, the respective refractive indices and extinction coefficients of different scatterers is shown in Figures S2b and S2c.^6–10^


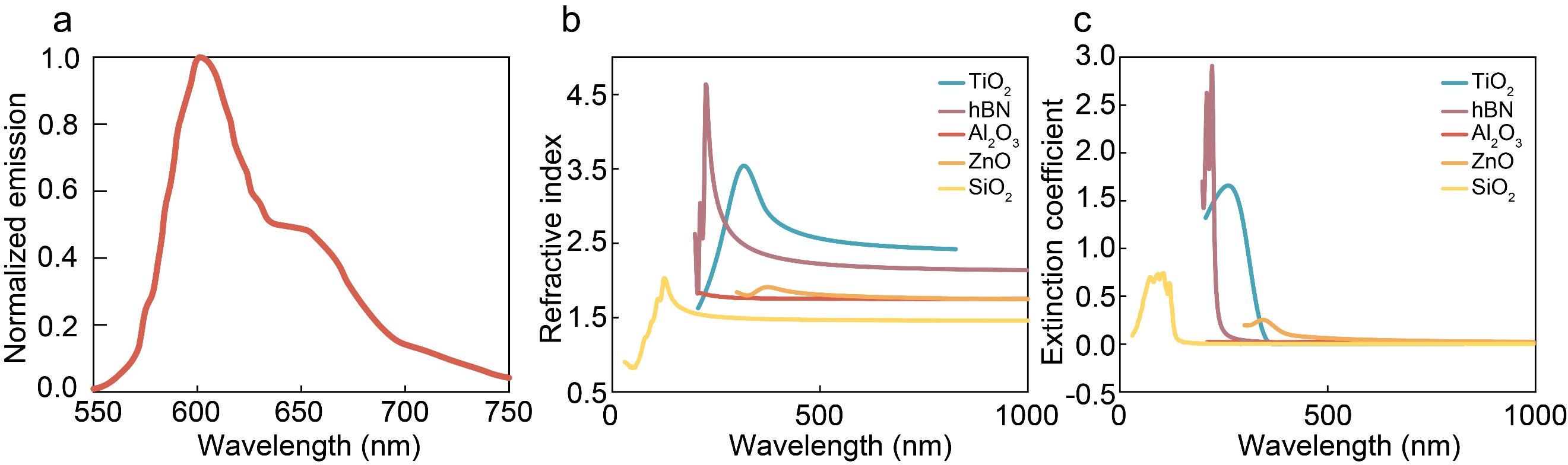


**Figure S2. Simulation spectra for light extraction efficiency in fluorescent films with scattering particles.** **(a)** Emission spectrum of commercial LF305 fluorescent powder used as the energy distribution profile for the volumetric emission source in the simulation. **(b)** and **(c)**Wavelength-dependent optical properties (refractive indices and extinction coefficients) of various scattering materials used to calculate their impact on light extraction efficiency.

Section 4 Fabrication schematic


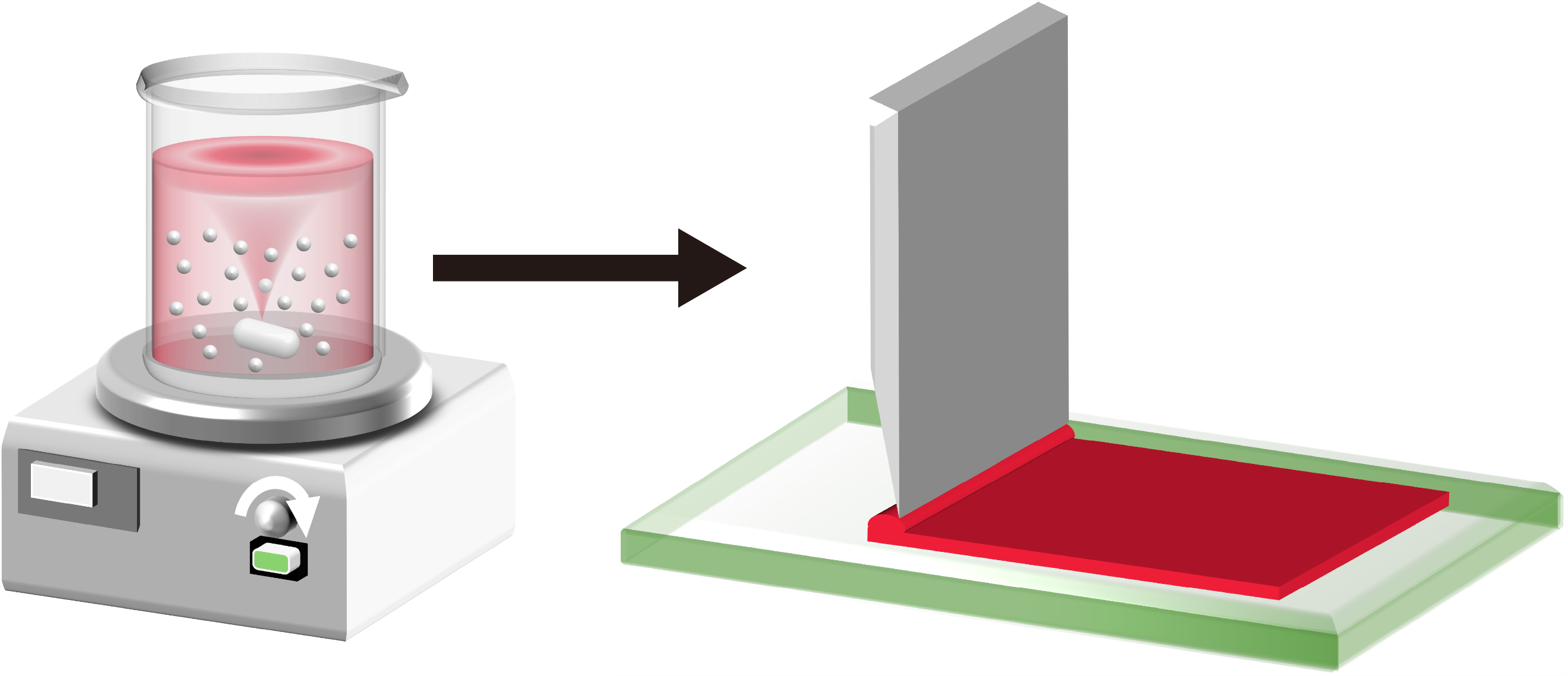


**Figure S3. Fabrication process of scattering-enhanced fluorescent films using blade coating.** The fabrication process includes the preparation of fluorescent polymer solutions by mixing LF305 fluorophore (0.1 wt% of total solute), TiO_2_ nanoparticles (0 wt%, 0.5 wt%, or 15 wt%), and PMMA in DMF solvent, followed by 24-hour magnetic stirring, thickness-control coating, and solvent evaporation.

Section 5 Different concentrations of TiO_2_


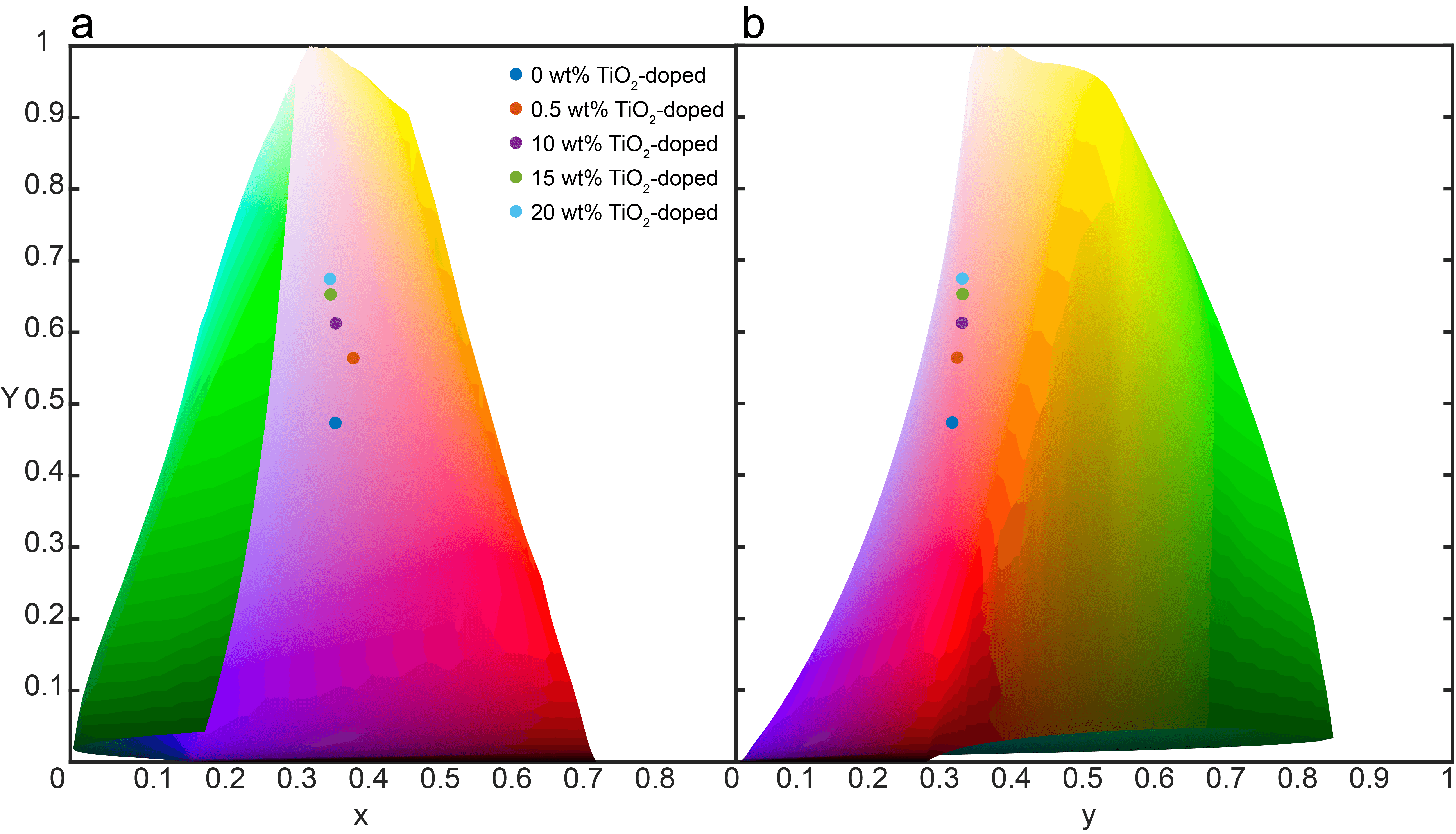


**Figure S4. Color variation of different concentrations (0, 0.5, 10, 15, 20 wt%) of fabricated TiO_2_-doped film. (a)** and **(b)** are front and side views of color coordinates of samples doped with different TiO_2_ concentrations in CIE Yxy space. The lightness of the fluorescent film undergoes a process of first increasing, then decreasing, and then increasing again with the increase of TiO_2_ concentration.


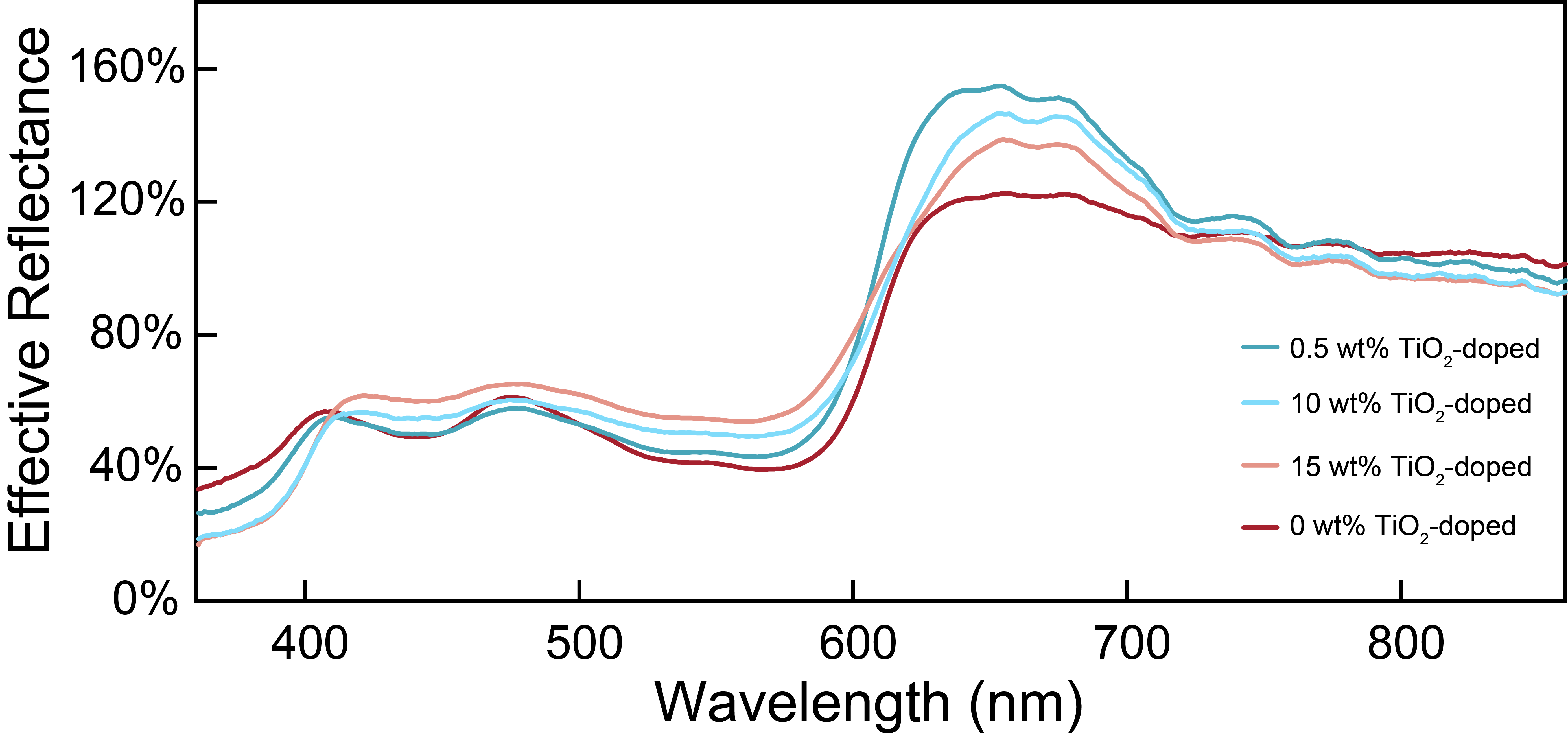


**Figure S5. Reflectance spectra of fabricated fluorescent films doped with varying concentrations of TiO_2_ (0, 0.5, 10, and 15 wt%).** In the fluorescence emission wavelength range, the reflectance peaks show a significant initial increase followed by a gradual decrease as the TiO_2_ doping concentration increases.

Section 6 EDS mapping for TiO_2_-doped fluorescent film


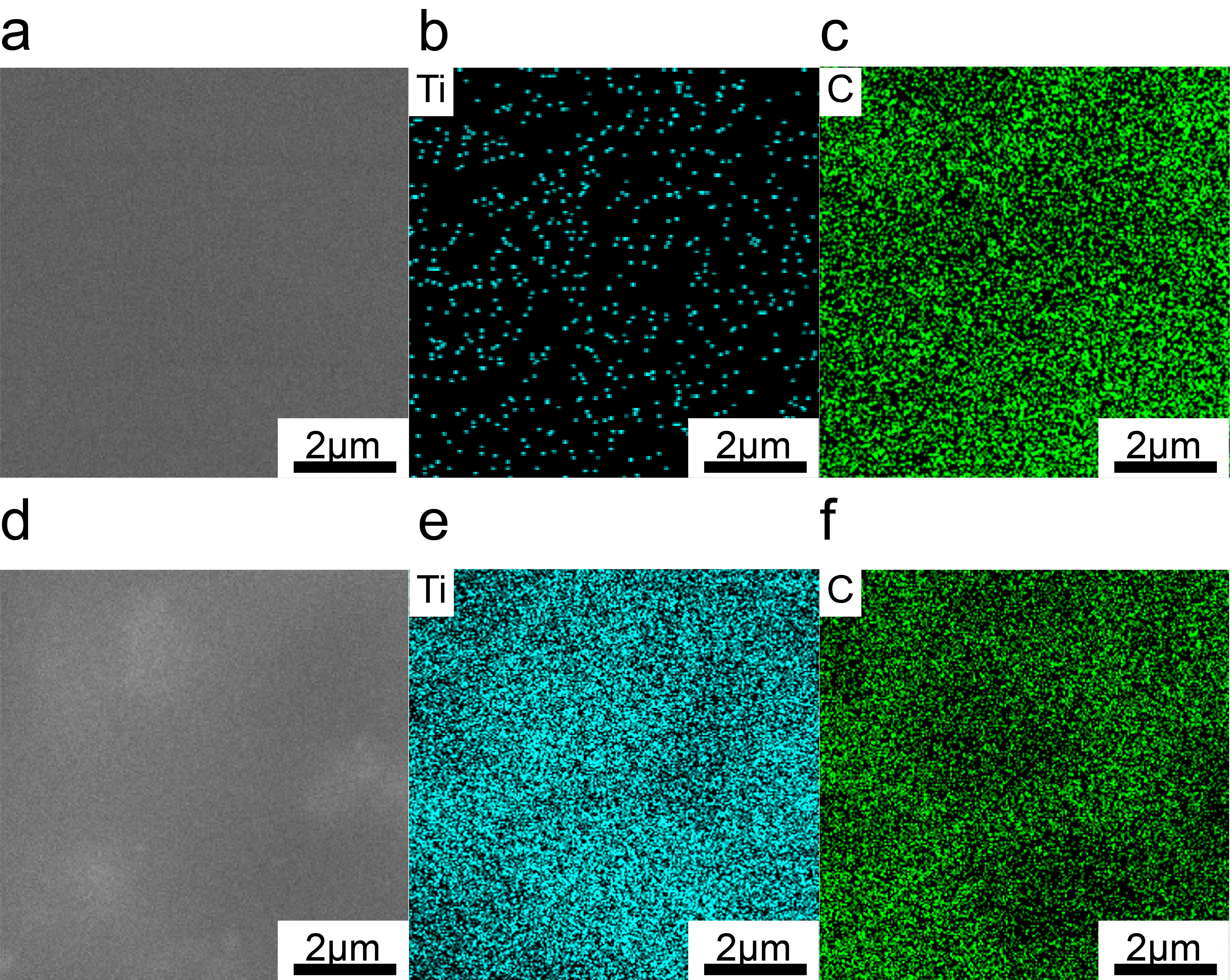


**Figure S6. EDS mapping demonstrates the uniform dispersion of TiO_2_ in fluorescent films doped with 0.5 wt% and 15 wt% TiO_2_.** (a)-(c) show the SEM image and the corresponding EDS maps of Ti and C for the fluorescent film doped with 0.5 wt% TiO_2_. (d)-(f) present the SEM image and the corresponding EDS maps of Ti and C for the fluorescent film doped with 15 wt% TiO_2_.

Section 7 Humidity record in outdoor experiment


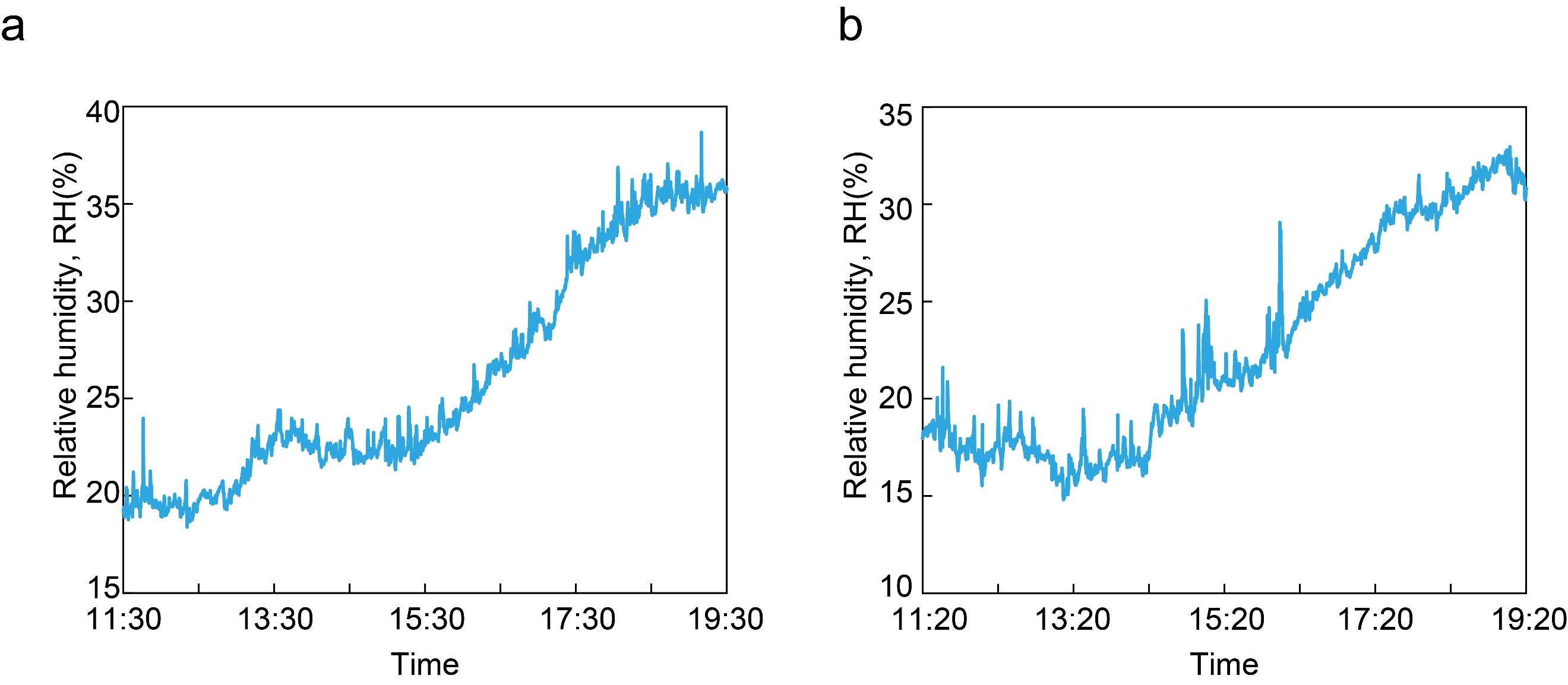


**Figure S7. Humidity variation curves of outdoor experiments in November (a) 28 and (b) 29, 2024.**

Section 8 Summer temperature record in outdoor experiment


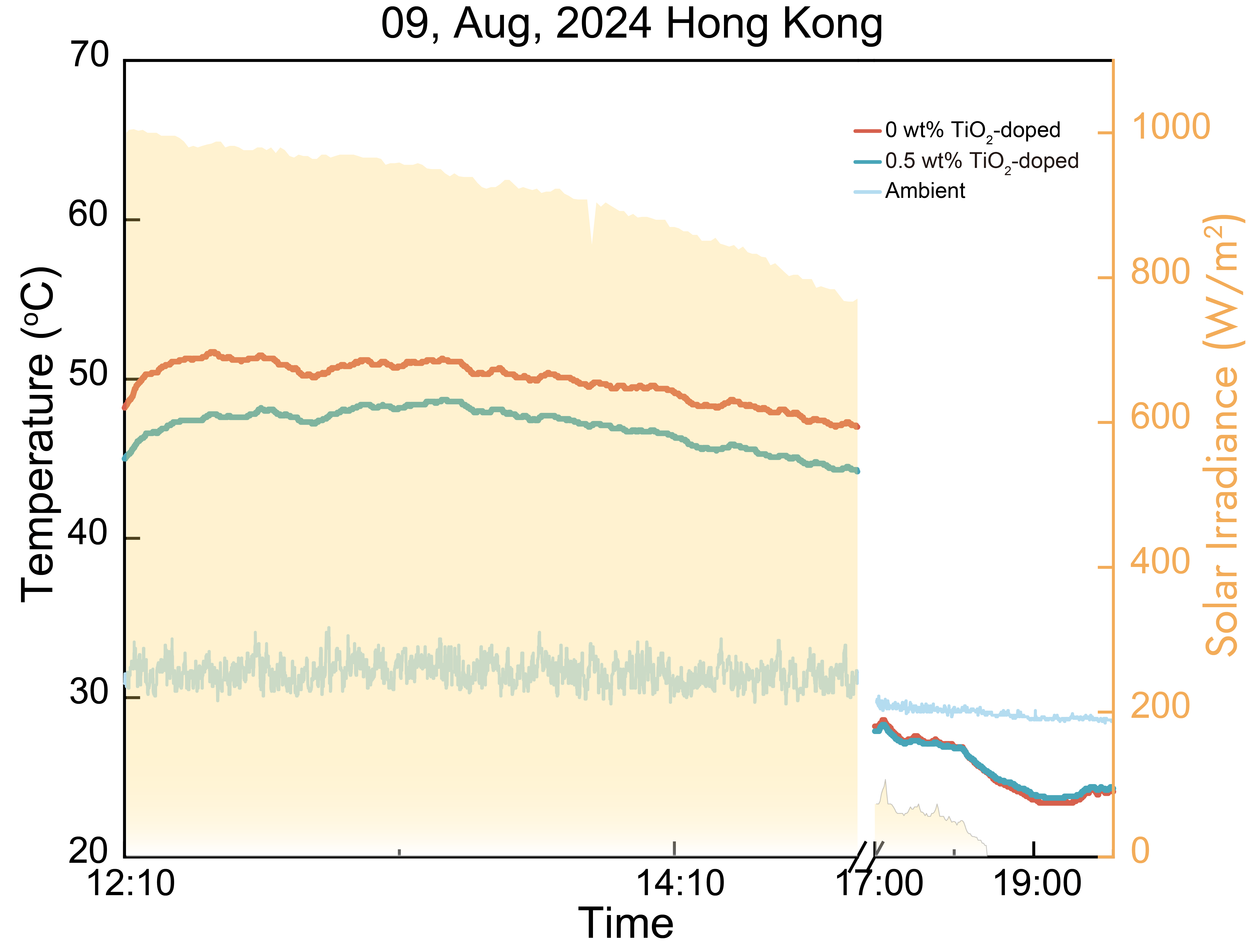


**Figure S8. Outdoor temperature monitoring of fluorescent films in August in Hong Kong.** The fluorescent film doped with 0.5 wt% TiO_2_ achieved an average temperature reduction of 2.9 °C and a maximum reduction of 4.1°C at noon compared to the undoped film. The ambient temperature at noon was approximately 31°C, with solar irradiance peaking at nearly 1000 W/m^2^.

# **References**

(1) Wright, W. D. A Re-Determination of the Mixture Curves of the Spectrum. *Transactions of the Optical Society* **1930**, *31* (4), 201.

(2) Wright, W. D. A Re-Determination of the Trichromatic Coefficients of the Spectral Colours. *Transactions of the Optical Society* **1929**, *30* (4), 141.

(3) Dieter Kraft. A Software Package for Sequential Quadratic Programming. *Forschungsbericht- Deutsche Forschungs- und Versuchsanstalt fur Luft- und Raumfahrt* **1988**.

(4) Jorge Nocedal, S. J. W. *Numerical Optimization*; 2006.

(5) Mcdowall, S. R.; Butler, T.; Bain, E.; Scharnhorst, K.; Patrick, D. Comprehensive Analysis of Escape-Cone Losses from Luminescent Waveguides. *Appl Opt* **2013**, *52* (6), 1230–1239.

(6) Jolivet, A.; Labbé, C.; Frilay, C.; Debieu, O.; Marie, P.; Horcholle, B.; Lemarié, F.; Portier, X.; Grygiel, C.; Duprey, S.; Jadwisienczak, W.; Ingram, D.; Upadhyay, M.; David, A.; Fouchet, A.; Lüders, U.; Cardin, J. Structural, Optical, and Electrical Properties of TiO2 Thin Films Deposited by ALD: Impact of the Substrate, the Deposited Thickness and the Deposition Temperature. *Appl Surf Sci* **2023**, *608*.

(7) Grudinin, D. V.; Ermolaev, G. A.; Baranov, D. G.; Toksumakov, A. N.; Voronin, K. V.; Slavich, A. S.; Vyshnevyy, A. A.; Mazitov, A. B.; Kruglov, I. A.; Ghazaryan, D. A.; Arsenin, A. V.; Novoselov, K. S.; Volkov, V. S. Hexagonal Boron Nitride Nanophotonics: A Record-Breaking Material for the Ultraviolet and Visible Spectral Ranges. *Mater Horiz* **2023**, *10* (7), 2427–2435.

(8) Aguilar, O.; de Castro, S.; Godoy, M. P. F.; Rebello Sousa Dias, M. Optoelectronic Characterization of Zn 1-x Cd x O Thin Films as an Alternative to Photonic Crystals in Organic Solar Cells . *Opt Mater Express* **2019**, *9* (9), 3638.

(9) Zhukovsky, S. V.; Andryieuski, A.; Takayama, O.; Shkondin, E.; Malureanu, R.; Jensen, F.; Lavrinenko, A. V. Experimental Demonstration of Effective Medium Approximation Breakdown in Deeply Subwavelength All-Dielectric Multilayers. *Phys Rev Lett* **2015**, *115* (17).

(10) Rodríguez-de Marcos, L. V.; Larruquert, J. I.; Méndez, J. A.; Aznárez, J. A. Self-Consistent Optical Constants of SiO2 and Ta2O5 Films. *Opt Mater Express* **2016**, *6* (11), 3622.
